# Childhood leukaemia incidence and the population mixing hypothesis in US SEER data

**DOI:** 10.1038/sj.bjc.6602432

**Published:** 2005-03-01

**Authors:** R C Parslow, G R Law, R G Feltbower, P A McKinney

**Affiliations:** 1 Paediatric Epidemiology Group, Centre for Epidemiology and Biostatistics, Leeds Institute of Genetics, Health and Therapeutics (LIGHT), University of Leeds, 30 Hyde Terrace, Leeds LS2 9LN, UK; 2 Epidemiology and Genetics Unit, Department of Health Sciences, University of York, Seebohm Rowntree Building, York YO10 5DD, UK

**Sir**,

We read with interest the paper by Wartenberg *et al* (2004) using the US SEER data, to examine the population mixing hypothesis in relation to the aetiology of childhood leukaemia. We have previously examined this issue in the UK using ecologic (Parslow *et al*, 2002) and case–control (Law *et al*, 2003) methodology. In their paper, Wartenberg and colleagues suggest that our ecologic study (Parslow *et al*, 2002) was of a similar type, and obtained similar results to those of Kinlen (1995). Unfortunately, this is not the case and our findings have been misinterpreted.

Firstly, our study design and methodology are different: Kinlen has found increased incidence of childhood leukaemia in areas that had unusually high levels of population movements, principally involving unusual sociodemographic events such as wartime evacuation (Kinlen and John, 1994) in relatively small populations. In contrast, our studies have used large populations determined *a priori* (either Great Britain as a whole or Yorkshire) and have not selected rural areas with sudden increases in population mixing (e.g. Kinlen and John, 1994; Kinlen, 1995).

Secondly, our investigations have used a well-defined, reproducible measure of population mixing. In addition, our exposure of interest, that is, population mixing, was derived from independently collected data sources. Our measure of population mixing is more comprehensive in comparison to crude changes in the size of a population as it includes an index of the diversity of inwards migration. The role of the diversity of migrants may contribute to the impact of population mixing on the level of circulating infections (Rhodes and Anderson, 1996).

Thirdly, our results indicate that high levels of population mixing confer a protective effect for childhood leukaemia (Parslow *et al*, 2002; Law *et al*, 2003). We have suggested that this may be due to early establishment of immunocompetence in areas of high population mixing and would support the second step in the delayed infection hypothesis (Greaves, 1997). This proposes that common-ALL is an unusual response to a common infection, following an initial chromosome translocation event *in utero*, as a result of limited exposure to childhood infections.

This misinterpretation of our study is not surprising: a plethora of papers exist in the literature that address the ‘population mixing hypothesis’. The definition of population mixing appears to vary by each investigation. Further research testing this hypothesis should provide a clear definition of the population mixing measure used.
